# Wnt target genes and where to find them

**DOI:** 10.12688/f1000research.11034.1

**Published:** 2017-05-24

**Authors:** Aravinda-Bharathi Ramakrishnan, Ken M. Cadigan

**Affiliations:** 1 Department of Molecular, Cellular and Developmental Biology, University of Michigan, Ann Arbor, MI, USA

**Keywords:** Wnt, beta-catenin, gene regulation, target location, T-cell factor

## Abstract

Wnt/β-catenin signaling is highly conserved throughout metazoans, is required for numerous essential events in development, and serves as a stem cell niche signal in many contexts. Misregulation of the pathway is linked to several human pathologies, most notably cancer. Wnt stimulation results in stabilization and nuclear import of β-catenin, which then acts as a transcriptional co-activator. Transcription factors of the T-cell family (TCF) are the best-characterized nuclear binding partners of β-catenin and mediators of Wnt gene regulation. This review provides an update on what is known about the transcriptional activation of Wnt target genes, highlighting recent work that modifies the conventional model. Wnt/β-catenin signaling regulates genes in a highly context-dependent manner, and the role of other signaling pathways and TCF co-factors in this process will be discussed. Understanding Wnt gene regulation has served to elucidate many biological roles of the pathway, and we will use examples from stem cell biology, metabolism, and evolution to illustrate some of the rich Wnt biology that has been uncovered.

## Introduction

The Wnt/β-catenin (Wnt/β-cat) pathway is conserved throughout metazoans and is essential for development and tissue homeostasis in adult organisms (reviewed in 1– 3). Aberrant Wnt/β-cat signaling is linked to several diseases, e.g. many cancers ^4, 5^ as well as bone and metabolic disorders ^6^. Intense investigation into the mechanisms of this pathway has uncovered some of the basics of how Wnts influence gene expression. A better understanding of how this signaling cascade operates has also provided genetic tools to explore various aspects of Wnt biology (reviewed in 7, 8). In addition, the identification of Wnt transcriptional targets has enhanced our knowledge of the biological importance of Wnt/β-cat signaling. In this short review, we will summarize recent findings on how the Wnt/β-cat pathway regulates transcription and provide examples of how identifying Wnt targets has broadened our knowledge of stem cell biology, the regulation of metabolism, and the evolution of physical traits.

**Figure 1.**
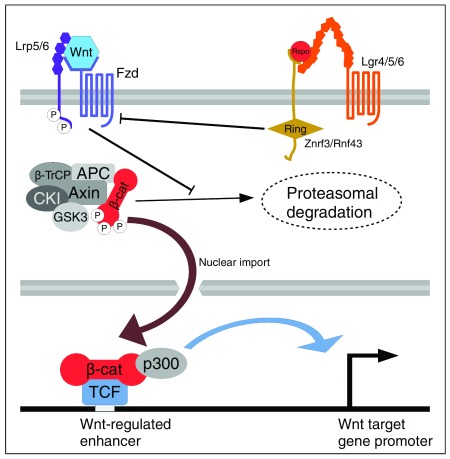
Overview of vertebrate Wnt/β-catenin (Wnt/β-cat) signaling. Wnt binding to Frizzled (Fzd) and low-density lipoprotein receptor-related protein 5/6 (Lrp5/6) co-receptors promotes the phosphorylation of Lrp5/6’s cytoplasmic tail. These interactions block the ability of the destruction complex to phosphorylate and ubiquitinate β-cat, preventing its degradation by the proteasome. Stabilized β-cat enters the nucleus, where it is recruited to Wnt-regulated enhancers by transcription factors (TFs) of the T-cell factor (TCF) family. R-spondin (Rspo) potentiates Wnt/β-cat signaling by increasing the number of Fzd receptors. Rspo forms a complex with Lgr4/5/6 and zinc and ring finger 3 (Znrf3)/ring finger protein 43 (Rnf43), preventing the latter from ubiquitinating Fzd receptors. APC, adenomatous polyposis coli; CKI, casein kinase I; GSK3, glycogen synthase kinase 3; β-TrCP, β-transducin repeat-containing E3 ubiquitin protein ligase.

The Wnt/β-cat pathway regulates the levels and subcellular localization of β-cat ( Figure 1). In unstimulated cells, β-cat is constantly degraded by a “destruction complex” containing the molecular scaffolds Axin and adenomatous polyposis coli (APC), the protein kinases glycogen synthase kinase 3 (GSK3)α/β and casein kinase I (CKI), and the ubiquitin E3 ligase β-transducin repeat-containing E3 ubiquitin protein ligase (β-TrCP) (reviewed in 9). Upon Wnt binding to a receptor complex containing Frizzled (Fzd) and low-density lipoprotein receptor-related protein 5/6 (LRP5/6), the destruction complex is inactivated, allowing the stabilization and nuclear import of β-cat (reviewed in 10). Nuclear β-cat is then recruited to chromatin by transcription factors (TFs), with members of the T-cell factor (TCF)/lymphoid enhancer-binding factor 1 (LEF1) family being the best characterized ^11– 15^ (reviewed in 16 and 17). In many cases, TCFs act as transcriptional switches, repressing Wnt targets in the absence of signaling, in part by the recruitment of transducin-like enhancer of split (TLE)/Groucho (Gro) co-repressors, which are displaced by β-cat, which then recruits co-activators such as the histone acetyltransferases CREB-binding protein (CBP) and p300 (reviewed in 18).

The pathway described above is highly conserved from sponges to humans ^1, 3^, but vertebrates have evolved a mechanism to regulate the sensitivity of cells to Wnt signaling. R-spondins (Rspos) are secreted proteins that potentiate Wnt/β-cat signaling (reviewed in 19). Rspos bind to two cell surface receptors, leucine-rich repeat-containing G-protein-coupled receptor (Lgr)4/5/6 and the E3 ubiquitin ligases zinc and ring finger 3 (Znrf3)/ring finger protein 43 (Rnf43) ^20, 21^. In the absence of Rspo, Znrf3/Rnf43 ubiquitinate Fzd receptors, targeting them for degradation ^20^. In this manner, Rspo signaling through Lgr and Znrf3/Rnf43 sensitizes the ability of cells to respond to Wnt signals by increasing the number of Fzd receptors ( Figure 1).

In the nucleus, TCF/β-cat can act through enhancers that can be hundreds of kilobases away from the proximal promoters of Wnt targets (e.g 22– 26). Enhancer-promoter communication can be explained by chromatin looping, and there was some prior evidence for this in Wnt gene regulation ^26– 28^. Jones and colleagues significantly extend these findings, demonstrating that Wnt-dependent looping occurs at multiple targets ^22^. Cohesin complexes are strongly associated with chromatin loops (reviewed in 29). Consistent with this, chromatin immunoprecipitation sequencing (ChIP-seq) was used to show a signal-dependent recruitment of cohesin subunits to Wnt-regulated enhancers ^22^. They also found that pathway activation does not greatly increase RNA polymerase II (Pol II) occupancy at promoters of Wnt targets, but it does increase phosphorylation of the C-terminal domain of Pol II, indicating that Wnt/β-cat signaling stimulates transcriptional elongation ^22^. This study provides the clearest description to date of some of the chromatin events that tie the binding of TCF and β-cat to enhancers with the initiation of transcription at Wnt target loci, and it will be interesting to see if they are typical for Wnt gene activation beyond the human embryonic stem cells used in this report.

## Updates to the standard model of Wnt gene regulation

The traditional assumption is that the recruitment of β-cat to chromatin results in transcriptional activation of nearby promoters (reviewed in 10, 18). However, a recent study systematically addressing this point found that the vast majority of β-cat binding sites in the chromatin of *Xenopus* gastrulating embryos had no detectable effect on gene expression ^30^. There was a strong overlap of the >10,000 β-cat ChiP-seq peaks identified in this report with TLE and p300 peaks from prior studies ^31, 32^, suggesting that many of these regions are functioning according to the standard model of Wnt-regulated enhancers. The authors propose a model of β-cat recruitment to regulatory DNA acting as a primer, with inputs from other signaling pathways required for activating transcription ^30, 33^. Interestingly, a priming role for β-cat has previously been proposed to occur at Wnt targets at an earlier developmental stage in *Xenopus*, prior to the onset of zygotic transcription at midblastula transition ^34^.

The work of Hoppler and colleagues highlights the challenges of using ChIP-seq to identify Wnt transcriptional targets. Another recent ChIP-seq/transcriptome analysis also found that only a small fraction of β-cat peaks were functional ^35^. The same is true when TCF peaks are matched to Wnt-regulated genes ^23, 36– 39^. But the study by Nakamura *et al*. ^30^ is interesting because it also considers p300 occupancy, which has a better track record of predicting functional enhancers ^40, 41^. That being said, even the most sophisticated models using multiple chromatin markers are still not perfect in locating functional enhancers ^42^. These studies highlight the complex nature of gene regulation and that clearly the recruitment of β-cat to chromatin is not sufficient for the activation of transcription.

Input from multiple signaling pathways on Wnt-regulated enhancers is one way to integrate information to precisely control gene expression, but cross-talk with other pathways can also occur outside the nucleus. Hippo signaling is a prominent example of cross-regulation with the Wnt/β-cat pathway that has received recent attention. Hippo signaling is an important regulator of cell proliferation and survival in animals (reviewed in 43, 44). A kinase cascade results in activation of the protein kinase large tumor suppressor kinase (LATS)1/2, which phosphorylates and inhibits the cytosolic proteins yes-associated protein (YAP) and tafazzin (TAZ). In the absence of LATS1/2 activity, YAP and TAZ translocate to the nucleus and serve as co-regulators for TEAD family TFs ^43, 44^. Initial reports found that YAP/TAZ inhibited Wnt/β-cat signaling ^45– 47^. In contrast to these reports, Piccolo and co-workers found that TAZ was targeted for degradation by the β-cat destruction complex ^48^. Wnt stimulation resulted in nuclear accumulation of both β-cat and TAZ, and a significant portion of the Wnt-induced transcriptional regulation was TAZ dependent in mammalian cell culture ^48^. Additional characterization revealed that YAP and TAZ were components of the destruction complex, which are dislodged upon Wnt stimulation ^49^. These authors provided evidence that Wnt-dependent YAP/TAZ release prevents β-TrCP association with the destruction complex, thus preventing β-cat degradation. Thus, YAP and TAZ can be viewed as integral components of the Wnt/β-cat signaling pathway in addition to their role in Hippo signaling ^49^.

Subsequent reports on the intersections between Hippo and Wnt/β-cat signaling support a complex and context-dependent relationship between the pathways. For example, LATS2 has been shown to directly inhibit β-cat’s interaction with other co-activators ^50^. YAP-dependent inhibition of Wnt/β-cat signaling has been reported in *Lgr5 ^+^* intestinal stem cells ^51^ and an antagonistic relationship between the Hippo and Wnt pathways was also observed in hepatocellular carcinomas ^52^. However, cooperation between the pathways consistent with the Piccolo model has been described during chronic inflammation-induced metaplasia in corneal epithelium ^53^. In addition, Wnt3a activates both TCF and TEAD reporters in skeletal muscle cells ^54^. Adding to the mechanistic insight linking YAP and β-cat, SET domain-containing lysine methyltransferase 7 (SETD7) is present in the destruction complex and methylates YAP, which is required for its ability to promote the nuclear accumulation of β-cat ^55^. Hippo and Wnt/β-cat signaling are connected through multiple mechanisms, and understanding the cell-specific cues that favor one interaction over another will be an important goal for future studies.

## The TCF transcriptional switch in vertebrates

Invertebrates such as *Drosophila* and *Caenorhabditis elegans* have one *TCF* gene, which plays a dual role on Wnt targets, inhibiting expression in the absence of signaling and mediating transcriptional activation when bound by β-cat (reviewed in 18). Vertebrates possess four or five *TCF* genes, with individual TCFs being more specialized, e.g. TCF3/TCF7L1 functions exclusively as a repressor ^56– 59^. In zebrafish, TCF3a and TCF3b repress Sry-related HMG box ( *Sox*) *4a* expression to inhibit spinal cord neurogenesis in a Wnt/β-cat signaling-independent manner ^60^. Recently, Merrill and co-workers reported a dramatic genetic interaction between *TCF3* alleles in mice that also supports a major role for β-cat-independent repression ^61^. *TCF3* null mutants die during gastrulation ^59^, while *TCF3* mutants lacking the β-cat binding domain ( *∆NTCF3*) die during late embryogenesis ^62^. Surprisingly, *TCF3 ^null^*/ *∆NTCF3* heterozygotes survive into adulthood with no obvious defects ^61^. This result demonstrates that TCF3 has an essential role in development that is independent of binding to β-cat.

How do some Wnt target genes undergo a transcriptional switch from repression by TCF3 to β-cat-dependent transcriptional activation by other TCFs? One model is that Wnt/β-cat signaling activates homeodomain-interacting protein kinase 2 (HIPK2), a kinase which phosphorylates TCF3, removing it from chromatin ^63, 64^. In mouse embryonic stem cells, several papers have reported a downregulation of TCF3 in backgrounds where Wnt/β-cat is elevated ^61, 65, 66^. Interestingly, the three reports differ on whether this effect acts at the level of transcription and/or post-transcriptionally. Under conditions in which mouse embryonic stem cells differentiate into endoderm, TCF3 downregulation coincided with elevated expression of the endodermal marker Forkhead Box A2 (FoxA2) and a loss of TCF3 on FoxA2 regulatory chromatin ^66^. Indeed, mouse embryonic stem cells lacking TCF3 have elevated FoxA2 expression and can differentiate into endoderm (albeit more slowly than normal) in the absence of Wnt stimulation ^66^. While this indicates that derepression of Wnt targets is a major driver for endoderm differentiation, another endoderm marker, Sox17, is directly activated by TCF4/TCF7L2 and β-cat ^67^. In sum, it appears as if derepression as well as β-cat activation of Wnt targets contribute to endoderm differentiation in vertebrates.

## TCFs and Wnt target location

All TCFs contain a HMG domain that can bind DNA in a sequence-specific manner (reviewed in 16). However, there is considerable degeneracy in the consensus binding site ^68^, to the degree that HMG-DNA recognition cannot be sufficient to drive TCF distribution on chromatin (reviewed in 1). This makes identifying Wnt targets solely through computational searches problematic ( Table 1). One way that some TCFs increase their DNA binding specificity is via a second domain, termed the C-clamp, located adjacent to the HMG domain and which binds GC-rich motifs called helper sites ^69^. The C-clamp is a novel Zn-binding domain ^70^ and C-clamp-helper site recognition is widely employed in Wnt target gene regulation in *Drosophila* and *C. elegans* ^70– 73^. In vertebrates, C-clamps are found in some isoforms of TCF1/TCF7L and TCF4/TCF7L2, where their presence extends the target selection of these TCF isoforms ^74, 75^.

**Table 1.** Approaches to identify Wnt target genes directly activated by the pathway. We define “direct Wnt targets” as genes whose regulatory DNA can be physically associated with T-cell factors (TCFs) or other transcription factors (TFs) and whose expression is modulated by the recruitment of β-catenin to regulatory chromatin by these TFs. The approaches outlined below each have their advantages and disadvantages, and a combination of them is required to establish with confidence that a gene is a Wnt target gene in a particular context.

Approach	Advantages	Disadvantages
**Computational searches for TCF binding sites** Position-weight matrices constructed based on validated lists of TCF binding sites can be used to screen *cis*-regulatory DNA for additional sites (e.g. 109). The efficiency of this approach can be improved by adding multiple sequences bound by TFs (e.g. helper sites in invertebrates; see 69). The functional relevance of binding sites can be verified with reporter assays.	• Quickly identifies potentially regulated genes • The identification of binding sites also establishes candidates for mutagenesis to rigorously test their functionality	• Most effective when the search space is restricted to short stretches of DNA (<20 kb) rather than the whole genome • Not all consensus TCF sites will be functional • TCFs and other TFs have degenerate binding sites that could be functional, which could be missed if the calling criteria are too stringent
**Transcriptome analyses of Wnt-regulated genes** Microarrays or RNA sequencing can be used to identify genes whose expression changes in Wnt-on and Wnt-off conditions in cell culture (e.g. 74) or embryos (e.g. 30)	• Identifies the full array of genes regulated by Wnt pathway activation • Many genetic and biochemical reagents are available to manipulate the Wnt pathway	• Does not distinguish between direct and indirect targets of Wnt signaling • *In vivo* analyses in animal tissues are limited by the specificity of the genetic drivers used for the manipulations
**Chromatin immunoprecipitation sequencing** **(ChIP-seq) analyses of TCF or β-catenin genomic** **occupancy** ChIP-seq with TCFs and β-catenin with or without Wnt activation can identify candidate Wnt-regulated enhancers. This approach can be combined with ChIP-seq for other TFs (e.g. 76) or with transcriptome analyses to assign genes to regulatory DNA sequences (e.g. 30).	• Biochemically establishes the presence of Wnt effectors at cis-regulatory elements • Provides evidence of direct regulation by the Wnt pathway	• Many TCF/β-catenin binding sites have no detectable function • Quality of the antibody used plays a major role • While this approach can identify putative Wnt-dependent cis-regulatory elements, identifying which gene the element regulates can be difficult, especially for long-range enhancers

Even in *Drosophila*, where there is one *TCF* gene containing a C-clamp, there is evidence that it associates with chromatin in conjunction with other TFs ^76^. Consistent with this, genome-wide surveys of TCF or β-cat binding in vertebrates reveal the presence of several TF binding site motifs besides the TCF site consensus ^1, 30, 39, 77, 78^. Other TFs have been reported to co-localize with TCFs ^36, 38, 39, 79, 80^, and in the cases of Cdx2 ^79^, Sp5/8 ^81^, and TEAD TFs ^82^, a co-dependency with TCFs or β-cat for chromatin association has been reported. In addition, TCF binding to chromatin is highly cell type specific ^36^ and is dynamic over time in the same cell type ^38, 39^. The data support a picture where different Wnt-regulated enhancers have different binding site grammars, which likely is a major mechanism by which Wnt/β-cat signaling regulates transcription programs in a cell-specific manner.

Are TCFs the major transcriptional mediators of Wnt/β-cat signaling in vertebrate systems? There are several TFs besides TCFs that can bind β-cat and regulate reporters in a β-cat-dependent manner (reviewed in 1, 10, 16), but information on their physiological relevance is limited. Identifying the β-cat binding domains on these TFs would provide valuable tools for investigating these interactions. For example, it is well known that deletion of the N-terminus of TCFs (∆NTCFs) results in potent dominant negatives ^16^. Expression of a ∆NTCF4 in colorectal carcinoma cells resulted in a reduction of >95% of the β-cat ChIP-seq peaks ^83^. One interpretation of these dramatic results is that TCFs are the predominant β-cat recruiters in these cells, at least under the experimental conditions used. These data do not rule out cooperation between TCFs and other TFs in β-cat recruitment and highlight the importance of generating TF mutants with specific defects in β-cat binding.

## Wnt target genes inform about stem cell biology

Wnt signaling is considered crucial for tissue maintenance by regulating stem cells in many tissues, and examining the expression of Wnt targets has been a successful strategy for identifying Wnt-regulated stem cell populations ^2^. Lineage-tracing approaches using knock-in alleles of Cre recombinase into the genomic loci of Wnt targets allows fate mapping of the progeny of Wnt-active stem cells. The first major success of this approach was the identification of stem cells in the small intestinal crypts. *Lgr5* was initially identified as a Wnt target in colon cancer cell lines ^84^. Subsequent *in vivo* analysis showed that its expression in the intestinal epithelium was limited to crypt base stem cells, and the ability of *Lgr5 ^+^* cells to give rise to epithelial cell types was confirmed through lineage tracing ^85^. A gradient of Wnt signaling has been demonstrated to be essential for the maintenance of *Lgr5 ^+^* intestinal stem cells, bolstering the idea of *Lgr5* as a Wnt target ^86^. *Lgr5* has since been shown to mark stem cell populations in the hair follicle ^87^, ovarian epithelium ^88^, and numerous other tissues ^89^. It is unclear whether it is a Wnt target in all cases.

A more widely expressed Wnt target is *Axin2*, whose expression domains resemble Wnt expression patterns ^90^. *Axin2* was first used for fate mapping in the mammary gland ^91^ and has recently been used to investigate the origin of liver cells. The polyploid nature of hepatocytes has long raised the question of whether they arise from cell division or by differentiation from a stem cell progenitor. The liver is divided into hexagonal lobules, each containing a central vein in the middle. A population of mostly diploid *Axin2*-expressing cells surrounds the central vein ^92^. Lineage tracing by fluorescently labeling *Axin2 ^+^* cells showed that they give rise to progeny that can be found throughout the lobule. Centrally located cells remain labeled, suggesting self-renewal ^93^. These results establish *Axin2 ^+^* cells as progenitors of polyploid hepatocytes.

In contrast to the stem cells of the intestine and liver, stem cells of the nail epithelium are seemingly agnostic to Wnt/β-cat signaling but require the pathway for differentiation. *Keratin-14* ( *K14*)-expressing cells located in the nail matrix were identified as nail stem cells (NSCs) through lineage tracing ^94^. A conditional knockout of β-cat in *K14 ^+^* cells impaired nail growth, with the entire nail epithelium showing elevated levels of NSC markers. Surprisingly, overexpressing a stabilized β-cat in *K14 ^+^* cells did not impact nail growth ^94^. In addition to its being a continuously growing tissue in adults, the nail epithelium has been studied for its role in digit tip regeneration. A population of *Wntless* ( *Wls*)-expressing cells—Wls is an acyltransferase required for the secretion of Wnt proteins ^95^—flanks the NSCs and is essential for digit tip regeneration. Digit tip regeneration does not happen after amputations that remove this population, but this defect can be rescued by the expression of β-cat in *K14 ^+^* cells. In this context, the Wnt/β-cat pathway appears to be a permissive signal that is essential for differentiation but has no influence on NSCs. Consistent with this, NSCs do not express high levels of Axin2 ^94^.

## Wnt target genes in metabolic regulation

The Warburg effect or aerobic glycolysis is seen in cancer cells, which preferentially metabolize glucose through lactic acid fermentation instead of the TCA cycle, even in the presence of oxygen ^96^. A colon cancer cell line with elevated Wnt/β-cat signaling expressing a ∆NTCF4 isoform containing a C-clamp showed reduced proliferation and a metabolic shift towards oxidative respiration. Consistent with this, genes controlling the cell cycle and metabolism were downregulated by this dominant negative TCF4 ^74, 97^. Interestingly, ∆NTCFs lacking a C-clamp did not affect proliferation but still caused the metabolic shift ^97^. Pyruvate dehydrogenase kinase 1 (PDK1), which blocks oxidative respiration, was found to be the key Wnt target promoting aerobic glycolysis in these cancer cells ^97^. PDK1 and the lactate transporter monocarboxylate transport protein (MCT)-1 are direct targets of the Wnt pathway in this context ^97, 98^ and lactate dehydrogenase, another enzyme driving the Warburg effect, was indirectly activated by the Wnt target *c-Myc* ^97, 99^. Interestingly, fluorescence lifetime microscopy (FLIM), which can provide an indicator of the relative rates of glycolysis and oxidative phosphorylation in live tissue, found that aerobic glycolysis also occurs in Wnt-dependent Lgr5 ^+^ intestinal stem cells ^97, 100^.

## Wnt target genes and animal evolution

In contrast to transcriptional profiling, Wnt targets that are important in animal evolution have been identified through linkage studies. The three-spined stickleback has become a premier system for studying the evolution of physical traits, since the marine species has repeatedly lost its body armor and ventral spines after colonizing freshwater lakes ^101– 103^. Characterization of marine/freshwater hybrids identified the *Ectodysplasin* ( *Eda*) locus as a major gene responsible for the loss of lateral armor plates in the freshwater species ^104^. Further refinement identified a single point mutation in an enhancer just downstream of *Eda* ^105^. This enhancer is a target of Wnt/β-cat signaling, and the freshwater allele has reduced activation ^105^. While this 3.2 kb enhancer contains several putative TCF binding sites, they are not close to the polymorphism (K. M. Cadigan, unpublished data), and it is not clear whether *Eda* is a direct target of the pathway. A similar story exists for the evolution of wing spots in *Drosophila guttifera*, where the *yellow* gene is activated by Wnt/β-cat signaling in the pupal wing, though the activation appears to be indirect ^106^.

Another link between Wnt/β-cat and evolution comes from a genome-wide association study (GWAS) which identified a polymorphism near the *KITLG* locus that is responsible for blond hair in humans ^107^. *KITLG* encodes a ligand for the KIT receptor, known to control pigmentation in mammals ^108^. Interestingly, the polymorphism resides in a predicted TCF binding site, with the blond allele showing reduced activation by Wnt/β-cat signaling ^109^. This study directly links the regulation of a direct Wnt target to an important physical trait. This is reminiscent of a polymorphism 335 kb upstream of the *c-myc* locus, also in a functional TCF site, where the higher-affinity allele is linked to increased risk in colorectal and other cancers ^26, 110^. Both examples illustrate how a detailed understanding of Wnt gene regulation can facilitate the molecular understanding of polymorphisms in the human population.

## Future directions

An increasing number of molecular approaches can now be employed to identify Wnt transcriptional targets in cells or tissues. Continued definition of Wnt transcriptional programs will further the understanding of how the Wnt/β-cat signaling pathway achieves its varied roles in development, stem cell maintenance, and metabolic regulation as well as in disease states and molecular evolution. It is clear that the activation of Wnt targets is highly context dependent, and the emerging picture is that a combination of TCFs and a diverse assortment of other TFs work together in different cells at different times. Unraveling the molecular mechanisms behind context specificity in Wnt responses will not only address a central question of gene regulation but also enhance our knowledge of the diversity of Wnt biology.

## Abbreviations

ChIP-seq, chromatin immunoprecipitation sequencing; *Eda*, *Ectodysplasin*; FoxA2, Forkhead Box A2; Fzd, Frizzled; *K14*, *keratin-14*; LATS, large tumor suppressor kinase; Lgr, leucine-rich repeat-containing G-protein-coupled receptor; NSC, nail stem cell; PDK1, pyruvate dehydrogenase kinase 1; Rnf43, ring finger protein 43; Rspo, R-spondin; Sox, Sry-related HMG box; TAZ, Tafazzin; TCF, T-cell factor; TF, transcription factor; TLE, transducin-like enhancer of split; β-TrCP, β-transducin repeat-containing E3 ubiquitin protein ligase; *Wls*, *Wntless*; Wnt/β-cat, Wnt/β-catenin; YAP, yes-associated protein; Znrf3, zinc and ring finger 3.
